# Supplementary material for: A distributed algorithm to maintain and repair the trail networks of arboreal ants
Source: Sci Rep. 2018 Jun 18;8:9297. doi: 10.1038/s41598-018-27160-3 (PMC6006367; doi:10.1038/s41598-018-27160-3)
Supplement: Supplementary file 1 — Supplementary Information [file 41598_2018_27160_MOESM1_ESM.pdf]

A distributed algorithm to maintain and repair the trail networks of arboreal ants
Supplementary Information

Arjun Chandrasekhar[1], Deborah Gordon[*2], and Saket Navlakha[*1]

[1]The Salk Institute for Biological Studies, Integrative Biology Laboratory, La Jolla, CA 92037 USA
[2]Stanford University, Department of Biology, Stanford, CA 94035 USA

[*]Corresponding authors: dmgordon@stanford.edu, navlakha@salk.edu

Comparison to additional non-linear algorithms

The RankEdge algorithm requires an ant arriving at a junction to rank-order all outgoing edges by weight before making a decision. Thus, we test RankEdge against five other, arguably simpler algorithms. We describe these algorithms here, and present results on all of them in tables in the main text. Overall, we find that RankEdge does the best job of simultaneously explaining field data and performing well in simulations, and so we primarily focus on the RankEdge algorithm in our main and supplementary figures.

- MaxEdgeA: With probability $1-q_{\text{explore}}$, choose randomly among edges tied for the highest weight. With probability q_{explore}, choose randomly among all other edges. This algorithm only needs to distinguish between edges with the highest weight and edges with any other weight.

- MaxEdgeB: With probability $1-q_{\text{explore}}$, choose randomly among edges tied for the highest weight. With probability q_{explore}, choose randomly among all edges. This algorithm only needs to distinguish only between edges with the highest weight and edges with any other weight.

- MaxEdgeC: With probability $1-q_{\text{explore}}$, choose randomly among edges tied for the highest or second highest weight. With probability q_{explore}, choose randomly among edges with less than the second highest weight. This algorithm needs only distinguish between edges with the highest or second highest weight and edges with any other weight.

- MaxWeighted: With probability $1-q_{\text{explore}}$, choose randomly among edges tied for the highest weight. With probability q_{explore}, choose randomly among all edges with probability proportional to the weight of the edge. This is a hybrid algorithm, using MaxEdgeA when not exploring, and using Weighted otherwise.

- Deneubourg [1]: From node u, choose edge (u, v) with probability:

$$\frac{(a + w(u,v))^{1/q_{\text{explore}}}}{\sum_{(u,v')\in E(G)} (a + w(u,v'))^{1/q_{\text{explore}}}},$$

  where we set $a = 1$ to constrain this model to two parameters (as in the other models). Low values of q_{explore} more strongly biases towards picking the highest weighted edge, and vice-versa. We find that the maximum likelihood value of q_{explore} is $0.78 < 1$, meaning the exponent is strictly greater than 1. Thus, the probability an edge gets chosen is not a linear exponent function of the weight. Note that under this model, as q_{explore} goes to 0, the probability of taking the highest weighted edge goes to 1.

Removing cycles when comparing chosen paths

Figure S1 shows that ants that take the top path are all using the same core path, even if some ants go around the loop more times than others. To account for this, we removed cycles when computing and comparing chosen paths. An alternative would be to consider each path that takes a different number of loops unique. This, however, is limiting. For example, two paths that exactly overlap except for the number of loops used would be considered equivalent to two paths that have zero edges in common. We wanted to avoid having to set an arbitrary threshold on how "similar" two paths need be before they are considered the same, and thus we simply removed all cycles from chosen paths.

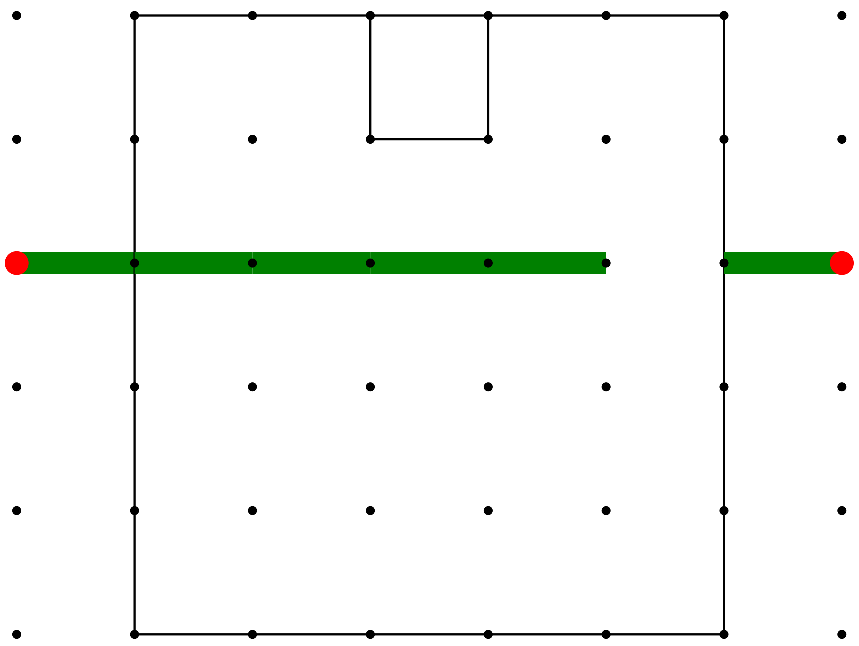

Figure S1: **Motivation for removing cycles.**

Variance of maximum likelihood estimations

The maximum likelihood heatmap in the main text (Figure 3) showed the cumulative likelihoods over all 13 turtle ant junctions. Here we confirm that the likelihood estimation produced consistent results in each of the 13 junctions.

We found the values for q_{explore} and q_{decay} that maximized the likelihood of producing the edge choices in each of the 13 junctions individually (Figure S2). We varied $q_{\text{explore}} \in [0.10, 0.40]$ and $q_{\text{decay}} \in [0.01, 0.30]$ in increments of 0.01 (the same range used for the maximum likelihood analysis in Figure 3), and recorded the maximum likelihood parameters for each junction. We then plotted a histogram of the distribution of the maximum likelihood parameters across the different junctions. Each maximum likelihood estimate is weighted by sample size; i.e., its contribution is weighted in proportion to the number of edge choices in that junction.

We observe that the distribution of most likely parameter values is tight, especially the values for q_{explore}, for which over 60% of the (weighted) junctions had nearly the same q_{explore} value (Figure S2). There is some variation in q_{decay}, possibly due to differences among days in environmental conditions that could affect pheromone evaporation rates. Overall, these results suggest that similar algorithm parameters apply for different colonies and days.

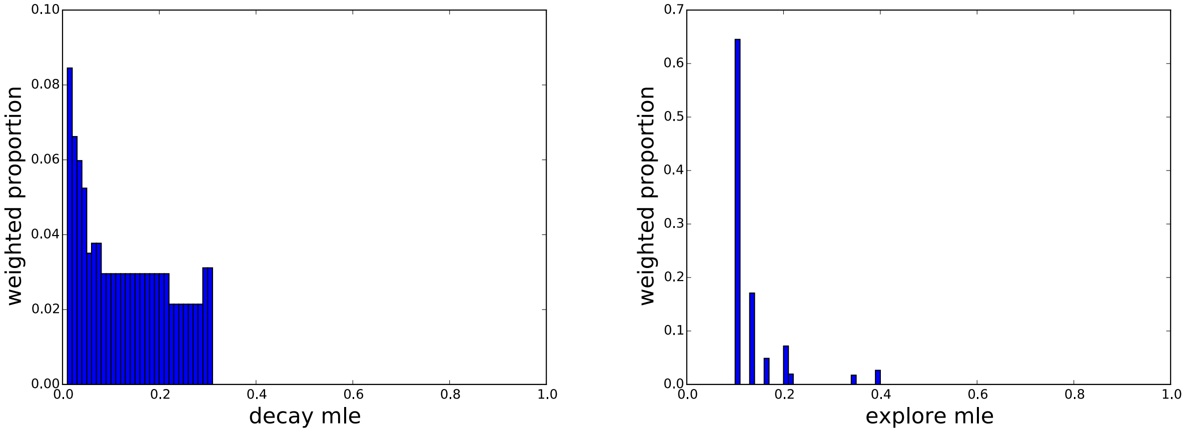

Figure S2: **Variance of maximum likelihood estimation.** Maximum likelihood analysis of the RankEdge algorithm for q_{decay} (left) and q_{explore} (right).

Comparing uni-directional vs. bi-directional search

Here we show that concurrent bi-directional search is crucial for the success of the algorithm. In initial simulations, ants are randomly distributed on the initial path, and ants from both sides of the break attempt to reach the other side.

To test the performance of an algorithm using uni-directional rather than bi-directional search, we performed simulations on the Full grid using RankEdge in which all ants were initialized to be on one side of the rupture. Figure S3 shows a significant loss in the ability to repair the network when using uni-directional search.

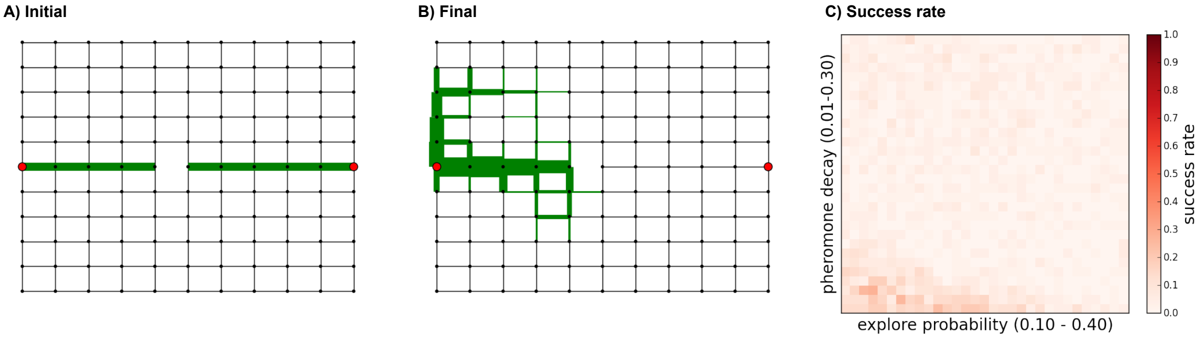

Figure S3: **Uni-directional vs bi-directional search.** A) Initial network. Simulations start with all ants placed at a random node on the left of the rupture. B) Final network. The pheromone on the right side quickly decays, making it much more difficult to build off the left path to repair the rupture. C) The success rate is significantly lower with uni-directional search than bi-directional search (main text).

Analyzing memory the power of avoiding backtracking

Our simulated ants are able to avoid revisiting the previously visited node, except when an ant reaches a nest and turns around to go back, or when it encounters a dead end.

Here we present results from simulations using the RankEdge algorithm with maximum likelihood parameter values, in which the ants are not prevented from using the previously visited node; i.e., they are allowed to backtrack. Figure S4 shows that under these conditions, performance is significantly worse than with one time-step of memory (see results for Full grid in the main text).

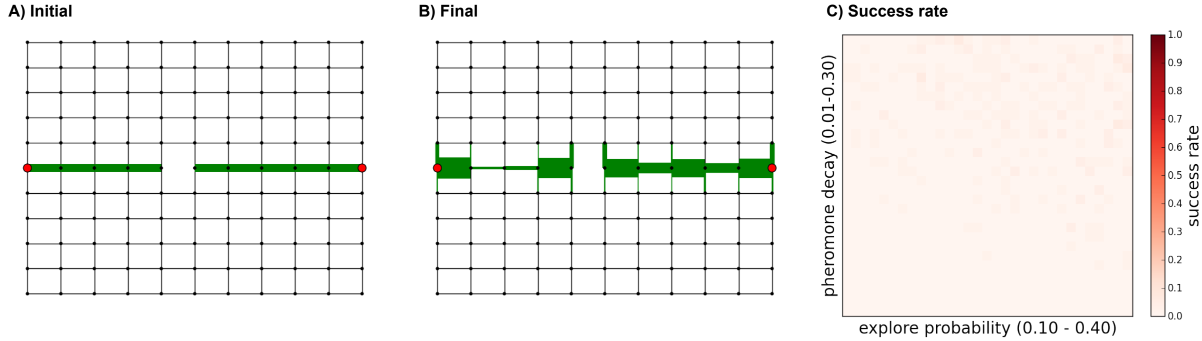

Figure S4: **Analysis of backtracking.** A) Initial network. B) Final network, showing that when ants are allowed to backtrack, few edges are repeatedly reinforced. C) The success rate is significantly lower when ants are allowed to backtrack (main text).

Performance on additional random graphs

Our analysis focused on planar graphs, since the turtle ant environment is a physical network. Here, we further test performance on two additional, well-studied random graph models: Watts-Strogatz [2] small-world networks ($n = 121, k = 4, p = 0.05$), and Erdos-Renyi (ER) random networks ($n = 121, p = 4/120$). The number of nodes n is equal to the number of nodes in the Full grid, and the other parameters were selected such that each node has an expected degree of 4 (also like the Full grid). We find that RankEdge has a success rate of 54% on ER networks and 46% on WS networks (geometric mean of 0.498), and a path entropy of 0 for both networks. By comparison, the Deneubourg algorithm achieved lower success rates of 46% on ER networks and 28% on WS networks (geometric mean of 0.359), also with path entropy of 0. Weighted, as expected, achieved 100% success but showed a very high path entropy: 6.69 on ER networks and 5.63 on WS networks.

The turtle ant algorithm evolved to operate on networks with physical, geometric constraints, whereas the ER and WS random network models are not constrained as such. For this reason we did not emphasize performance on these random networks. Further work is needed to investigate the theoretical relationship between the performance of RankEdge and network topology, as defined by properties such as degree distribution, clustering coefficient, or distribution of path lengths.

Performance when multiple links are broken

In field experiments and in the simulations reported in the main text, we ruptured only one link in the path. In the field, edges or stems are broken occasionally by the wind or an animal moving through the vegetation. It is extremely unlikely for two or more such events to occur concurrently in the same path, and the repair process for the repair of a single link is quite rapid [3]. Nonetheless, here we test performance of the algorithm with multiple broken links.

Specifically, we tested RankEdge on the Full grid with different numbers of broken links; starting from one edge ruptured to the entire path missing (Figure S5A). We achieved success rates of 70%, 42%, and 24%, 24%, and 0%, respectively (Figure S5B). The performance of the algorithm decreases the more links are broken. These results, as well as field observations, suggest that if many links are broken (e.g., the last panel in Figure S5A), a different search strategy is used. This strategy may be similar to the one used when turtle ants initially establish a new path connecting nests; this problem requires further study.

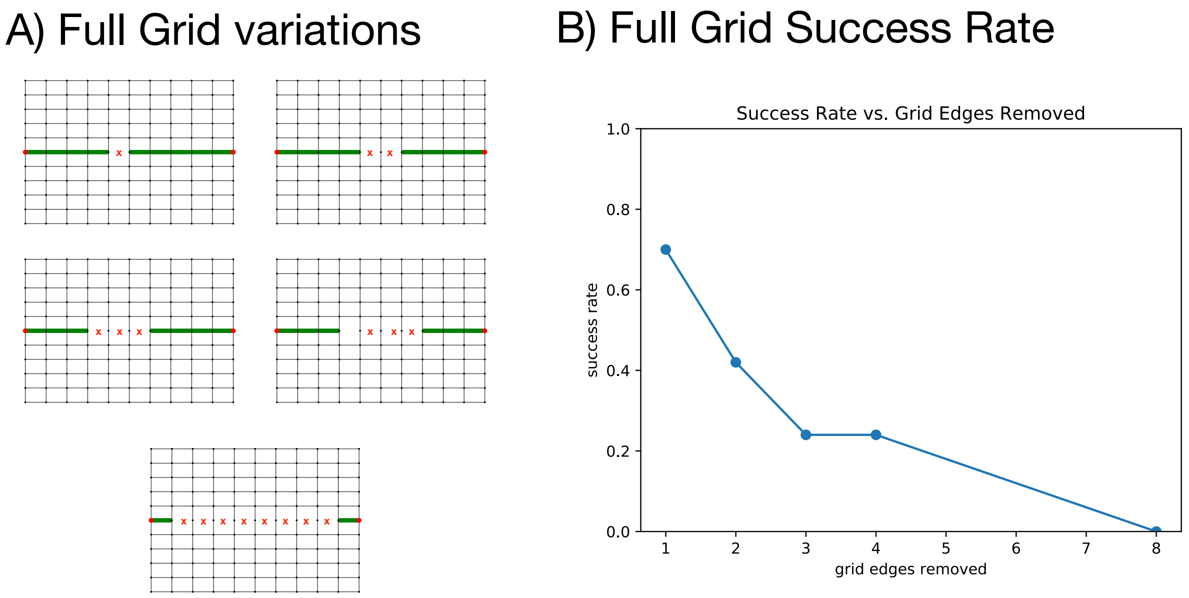

Figure S5: **Performance when multiple links are broken.**

Theoretical results

Here we provide a probabilistic argument for why the RankEdge algorithm prunes a dead-end significantly faster if ants do not lay pheromone on the way back from a dead-end. We do this by providing a coarse upper bound on the average time to prune in the two scenarios: when ants do not lay pheromone on the way back from a dead-end versus when they do lay pheromone on the way back.

Consider the Minimal graph, and the junction on the left where the ant must choose between the edge that leads to the nest on the right (the 'good edge'), and the edge that leads to the dead-end (the 'bad edge'). The RankEdge algorithm is biased towards selecting the highest weighted edge; therefore, for the ants to find the alternative path, there must be more pheromone on the good edge than on the bad edge.

Proof idea: Exponential decay means that the difference between the two edge weights is mostly determined by the most recent choices (i.e., recent ant choices have disproportionately more effect on the difference in the two edge weights than choices made long ago). Thus, for the good edge to overtake the bad edge in edge weight, it suffices for the ants to explore the good edge more than expected by chance in a short period of time. The ants can take advantage of high variability in small sample sizes to put more pheromone on the good edge than the bad edge, at which point the RankEdge algorithm will be biased towards selecting the good edge over the bad edge. Finally, by depositing only half as much pheromone on the bad edge than on the good edge (for reasons explained below), the ants reduce the number of explore steps needed in a short time period to put more pheromone on the good edge than the bad edge. We formalize this intuition below.

Proof: We make the following assumptions in the course of the proof:

- An ant makes a choice at the junction. If it takes the good edge, it will go over the edge and back in one step (Methods), or it will take the bad edge, and eventually come back to the junction after returning from the dead end. It will not lay pheromone on the way back from the dead-end.
  - No backtracking means that the ant might walk all the way to the dead end before turning around and discontinuing pheromone deposition. It will still lay only one unit of pheromone on the edge at the junction that leads to the dead end trail.
- We split time into intervals of K steps. For simplicity, we ignore decay in the current interval, consider choices with decay in the immediately previous interval, and ignore all choices in any prior intervals assuming all pheromone previously deposited has decayed so has no significant impact on the current weights.
- For simplicity, we assume an infinite number of ants; thus, after an ant chooses an edge and lays pheromone, it is put at the end of an infinite queue at node 1. This allows us to ignore the case in which an ant chooses the bad edge, and later is forced to choose the good edge due to the rule against backtracking. While this is obviously not how the simulations actually work, this simplifying assumption allows us to derive the intuition for how the algorithm works without getting bogged down in technical details. The RankEdge's 100% success on

the minimal network shows that this assumption does not cause an overbearing discrepancy between our theoretical analysis and the true nature of the simulations.

Informally, consider an ant at the junction that chooses between the good or the bad edge. The choice the ant makes at this junction determines whether it will reach the nest on the right side of the break or reach the dead-end. Thus, it suffices to consider a simplified version of the Minimal graph by considering only the choice made by the ant at that junction before the good and the bad edge. We seek to determine the probability and expected time needed for the ants to place more pheromone on the good edge than the bad edge.

As described in Methods, if an ant takes an "explore step" and chooses the lower weighted edge, it will deposit two units of pheromone on that edge. If an ant chooses the good edge on a non-explore step, it will leave one unit of pheromone on the way to the nest and one unit on the way back, so that edge will be reinforced with 2 units of pheromone. If an ant chooses the bad edge on a non-explore step, it will not deposit pheromone on the way back from the dead-end, so that edge will receive only 1 unit of pheromone. We will consider the case where the bad edge initially has more weight than the good edge, and we want to determine how much time it takes before the good edge has more weight than the bad edge. Once this happens, the ants will have formed a maximal path between the two nests.

Formally, we have a graph with nodes $V = \{1, 2, 3\}$ and edges $E = \{(1, 2), (1, 3)\}$. Edge $(1, 2)$ represents the bad edge (with initially higher pheromone), and $(1, 3)$ is the good edge. Additionally, let $W_t(u, v)$ to be the weight of edge (u, v) at time t. At each time-step, an ant at node 1 must choose between the two edges. The ants follow the RankEdge algorithm, so they take the higher weighted edge with probability $1 - q_{\text{explore}}$ and the lower weighted edge with probability q_{explore}. If the ant chooses edge $(1, 2)$ then it augments the weight of that edge by 1:

$$W_{t+1}(1, 2) \leftarrow W_t(1, 2) + 1.$$

If it chooses edge $(1, 3)$ then it augments that edge weight by 2:

$$W_{t+1}(1, 3) \leftarrow W_t(1, 3) + 2.$$

If the two edges have equal weight then it chooses each of the two with equal probability, but for now we will ignore that case.

At the end of each time-step each edge weight decays exponentially by a rate, q_{decay}:

$$W_{t+1}(1, 2) \leftarrow W_{t+1}(1, 2) \times (1 - q_{\text{decay}})$$

and

$$W_{t+1}(1, 3) \leftarrow W_{t+1}(1, 3) \times (1 - q_{\text{decay}}).$$

For example, suppose $W_t(1, 2) = 10$ and $W_t(1, 3) = 5$. With probability q_{explore}, the ant takes edge $(1, 3)$ and with probability $1 - q_{\text{explore}}$ it takes edge $(1, 2)$. If it takes $(1, 3)$ then

$$W_{t+1}(1, 3) \leftarrow W_t(1, 3) + 2 = 7,$$

and if it takes edge $(1, 2)$ then

$$W_{t+1}(1, 2) \leftarrow W_t(1, 2) + 1 = 11.$$

For example, suppose the ant takes edge $(1,3)$. Then,

$$W_{t+1}(1,2) \leftarrow 10, \quad W_{t+1}(1,3) \leftarrow 7.$$

At the end of the time-step, both edges decay by q_{decay}:

$$W_{t+1}(1,2) \leftarrow W_{t+1}(1,2) \times (1 - q_{\text{decay}}) = 10 \times (1 - q_{\text{decay}})$$

and

$$W_{t+1}(1,3) \leftarrow W_{t+1}(1,3) \times (1 - q_{\text{decay}}) = 7 \times (1 - q_{\text{decay}}).$$

Assume $W_0(1,2) > W_0(1,3)$; i.e., there is more pheromone on the bad edge than the good edge.

Let $X_0 = W_0(1,3) - W_0(1,2) < 0$, and $(X_t)_{t=1}^{\infty}$ be a sequence of independent and identically distributed (i.i.d.) random variables representing the difference between the amount of pheromone at time t contributed to the good edge minus the bad edge. Then, for $t \in \{1,2,3,\dots\}$:

$$\Pr(X_t = -1) = 1 - q_{\text{explore}}$$

and

$$\Pr(X_t = 2) = q_{\text{explore}}.$$

If $X_t = -1$ then the ant chose the bad edge and deposited 1 unit of pheromone on edge $(1,2)$. If $X_t = 2$ then the ant chose the good edge and deposited 2 units of pheromone on edge $(1,3)$. We want to determine when $W_t(1,3) > W_t(1,2)$.

To determine the difference in pheromone at any time t, we first need to determine how much pheromone from choices made at times $< t$ still remains. If an ant made a choice at time i, then due to exponential decay, at time $t > i$, the contribution of that choice (X_i) to the difference in weight between the good and bad edge is:

$$X_i \cdot (1 - q_{\text{decay}})^{t-i}.$$

And thus,

$$W_t(1,3) - W_t(1,2) = \sum_{i=0}^{t} X_i \cdot (1 - q_{\text{decay}})^{t-i}.$$

Let T represent the first time when there is more pheromone on the good edge than the bad edge, i.e.:

$$T = \inf \left\{ t : \sum_{i=0}^{t} X_i \cdot (1 - q_{\text{decay}})^{t-i} > 0 \right\}$$

Then at time T,

$$W_T(1,3) - W_T(1,2) > 0 \iff W_T(1,3) > W_T(1,2).$$

Thus we are interested in finding $E[T]$ to determine when the probability of taking the good edge is greater than that of taking the bad edge.

Informally, we want to determine how far back in time we need to consider choices before their contribution to the current difference in weight between the good and the bad edge becomes negligible. Consider a choice made at time nK, where K is some integer greater than 0. We want

to determine the value of K such that the difference in weight at time nK depends only on the choices made between times $(n-1)K$ to nK and negligibly on choices made prior to $(n-1)K$. We also want K to be such that, regardless of the choices made prior to nK, it is possible for the good edge to have more weight than the bad edge by time $(n+1)K$. Thus, we want a large enough K to ignore most past choices, but small enough to make significant deviations possible within the next K steps.

Formally, let $K \in \mathbb{N}$, and consider times $t = \{K, 2K, 3K, \ldots\}$. If K is sufficiently large such that $(1-q_{\text{decay}})^{2K} \approx 0$, then to compute the difference at time $(n+1)K$:

$$\begin{aligned}\sum_{i=0}^{(n+1)K} X_i \cdot (1-q_{\text{decay}})^{(n+1)K-i} &= \sum_{i=0}^{(n-1)K} X_i \cdot (1-q_{\text{decay}})^{(n+1)K-i} + \sum_{i=(n-1)K}^{(n+1)K} X_i \cdot (1-q_{\text{decay}})^{(n+1)K-i} \\ &= \sum_{i=0}^{(n-1)K} X_i \cdot (1-q_{\text{decay}})^{2K+(n-1)K-i} + \sum_{i=(n-1)K}^{(n+1)K} X_i \cdot (1-q_{\text{decay}})^{(n+1)K-i} \\ &\approx \sum_{i=(n-1)K}^{(n+1)K} X_i \cdot (1-q_{\text{decay}})^{(n+1)K-i}\end{aligned} \tag{1}$$

Taking $K = 75$ and $q_{\text{decay}} = 0.02$ (the RankEdge maximum likelihood estimate of q_{decay}), we see that

$$(1-q_{\text{decay}})^{2K} < 0.05,$$

which is sufficiently small such that we may approximate $W_t(1,3) - W_t(1,2)$ using Equation (1). Thus, due to exponential decay, at time nK, we can ignore X_m for $m < (n-1)K$.

We have now established that we need to consider only the choices made between times $(n-1)K$ to nK. Consider the worst-case scenario, where all choices made in this interval reinforce the bad edge. Specifically, $X_m = -1$ for all $(n-1)K \leq m < nK$. We next want to determine how much pheromone needs to be placed on the good edge in the next K steps to flip the probabilities. At time $(n+1)K$, the contribution of the choices made in this interval will be at least:

$$a = \sum_{m=(n-1)K}^{nK} -1 \cdot (1-q_{\text{decay}})^{(n+1)K-m} \leq \sum_{m=(n-1)K}^{nK} X_m \cdot (1-q_{\text{decay}})^{(n+1)K-m}$$

Thus, to flip the probabilities within the next K steps, the ants need to make enough contribution to the good edge such that:

$$\sum_{i=nK}^{(n+1)K} X_i \cdot (1-q_{\text{decay}})^{(n+1)K-i} > a.$$

Let S represent the number of times the ants chose the good edge in the interval $nK \leq t < (n+1)K$, i.e.:

$$S = |\{t : nK \leq t < (n+1)K, X_t = +2\}|$$

Let $(K - S)$ represent the number of times the ants chose the bad edge in this interval. Then, in the worst case, the number of times the good edge needs to be taken compared to the bad edge to flip the probabilities is:

$$\begin{aligned} 2S - (K - S) &\geq |a| \\ S &\geq \frac{K + |a|}{3}. \end{aligned}$$

Thus, the probability that the edges flip in the next K steps is at least:

$$\Pr(nK \leq T \leq (n+1)K) \geq \Pr\left(S \geq \frac{K + |a|}{3}\right).$$

The right-hand side of the equation above is approximately binomial with parameters $p = q_{\text{explore}}, n = K$, which can be further approximated by the normal distribution with parameters $\mu = q_{\text{explore}}, \sigma^2 = \frac{q_{\text{explore}}(1 - q_{\text{explore}})}{K}$. Note that in deriving S we ignored exponential decay between times nK and $(n+1)K$, so that we may treat the X_i in a small window of time as i.i.d. and apply a binomial approximation. It is not obvious how to compute $\Pr(nK \leq T \leq (n+1)K)$ with exponential decay included. Below, we find that this binomial approximation is still sufficient to provide an upper bound, and then we illustrate why it is crucial that the ants do not lay pheromone on the way back from the dead-end.

Taking $q_{\text{explore}} = 0.20, K = 75$ we get that:

$$\Pr\left(S \geq \frac{K + |a|}{3}\right) \approx 0.00011$$

Thus, the probability that the edges flip probabilities within nK and $(n+1)K$ is:

$$\Pr(nK \leq T \leq (n+1)K) \geq 0.00011.$$

If we think of each block of K steps as an independent trial with success probability $p \geq 0.00011$, then we can think of T as an approximately geometric random variable with parameter $p \geq 0.00011$. The expected value of T is then: $E[T] \leq K \cdot (1/p) \approx 75 \cdot 9104 = 682800$.

Informally, the exponential decay essentially gives the process an approximately memoryless property by nearly erasing the results of decisions that were sufficiently far in the past. To succeed, the ants simply need one block of K steps in which they take edge $(1, 3)$ more often than expected to push $W_t(1, 3)$ above $W_t(1, 2)$ and flip the probabilities. Note that this is the worst case possible, i.e., when the previous 75 time-steps all reinforce the bad edge, which is itself highly unlikely.

Next, we show that if the ants lay pheromone on the way back from the dead-end, then the expected value of T is significantly higher. Formally, if we eschew the condition that ants do not lay pheromone on the way back from a dead-end, then both the good and the bad edges increase their edge weight by 2 when traversed. Then the number of times S the ants need to chose the good edge in the interval $nK \leq t < (n+1)K$ is:

$$\begin{aligned} 2S - 2(K - S) &\geq a \\ S &\geq \frac{a + 2K}{4} \end{aligned}$$

For $q_{\text{explore}} = 0.20, q_{\text{decay}} = 0.02, K = 75$, we get that

$$Pr\left(S \geq \frac{2K + |a|}{4}\right) \approx 6.17 \cdot 10^{-13}$$

and

$$E[T] \leq K \cdot \frac{1}{p} = \frac{75}{6.17 \cdot 10^{-13}} \approx 1.22 \cdot 10^{14}.$$

Thus, the number of successes the ants need in a span of K steps is significantly higher, and so the chances of any one block of K steps flipping the probabilities is much lower and takes much more time.

In the unlikely event that the two edges ever have equal weight, the ant picks between the two edges with equal probability. With probability 0.5 it takes the good edge at which point the good edge has higher weight, and thus the break is fixed. With probability 0.5 the ant takes the bad edge at which point all of the above analysis may be repeated.

We performed 50 simulations of 1000 steps on the Minimal graph in which ants do lay pheromone on the way back from the dead-end. We find a 0% success rate, supporting our theoretical insights.

Necessity of queueing

The theoretical argument above provides intuition for why queuing is necessary for pruning a dead-end. The smaller the sample size, the more likely that enough choices will deviate from expectation toward the good edge to flip the probability toward taking the good edge. When ants cue, this decreases the sample size because fewer ants make a choice in each time step. This decrease in sample size then increases the probability that in a given time interval, ants choose the good edge over the bad edge. When ants do not queue, they make more choices in each time step, and so more time is required to achieve the deviation toward choosing the good edge over the bad edge. If there is no queuing, then initially only a fraction of ants are at the dead end, and the sample size argument above does not necessarily hold at the junction. Eventually however, all of the ants in the graph eventually approach the junction between the good and bad edge, to the point where our sample size argument above eventually takes hold.

Formally, suppose there are 100 ants. If there is no queuing, then 100 ants will make a choice at each time step, rather than 1 ant. If we follow the argument above, S would still be approximately normally distributed, but its variance would be $\frac{q_{\text{explore}}(1 - q_{\text{explore}})}{100K}$, and thus $\Pr\left(S \geq \frac{K + |a|}{3}\right)$ becomes much smaller because S is much more tightly distributed around its mean. This theoretical intuition is confirmed by simulation using 1000 steps. If the queuing mechanism is removed, there is a 0% success rate on the Minimal graph.
